# Head‐to‐head: Anthropomorphic phantoms for evaluating the effect of skin tone on the accuracy of surface imaging systems

**DOI:** 10.1002/acm2.70188

**Published:** 2025-07-15

**Authors:** Elizabeth L. Covington, Dennis N. Stanley, Ashanti Lawson, Scott Hadley, Alexander Moncion, Rodney J. Sullivan, John B. Fiveash, Richard A. Popple

**Affiliations:** ^1^ Department of Radiation Oncology The University of Alabama at Birmingham Birmingham Alabama USA; ^2^ Department of Radiation Oncology University of Michigan Ann Arbor Michigan USA

**Keywords:** intrafraction motion management, radiosurgery, SRS, surface imaging

## Abstract

**Purpose:**

To study the localization accuracy of two commercial surface imaging (SI) systems used for intrafraction motion monitoring during stereotactic radiosurgery (SRS) and quantify the difference in accuracy due to skin tone.

**Methods:**

Using a publicly available CT dataset, anthropomorphic phantoms were 3D printed using filament in four skin tones: rose tan, light brown, medium brown, and dark brown (3DUniverse, Chicago, IL, USA). Three SI systems from two vendors were utilized to measure the SI‐reported offsets of the stationary phantoms in various gantry and couch configurations to create SRS‐like conditions, including when the gantry obscured one camera pod. At each position, approximately 5 s of offsets were averaged to obtain the SI‐reported offset.

**Results:**

All SI systems reported larger offsets for all phantoms at non‐zero couch angles. The medium brown and dark brown phantoms showed the largest magnitudes at non‐zero couch angles, increasing further when a camera pod was obstructed. For two SI systems, the position of the phantom for medium and dark brown phantoms could not be resolved at certain gantry/couch positions, and offsets up to 5 mm were reported.

**Conclusion:**

Characterization of SI systems using 3D‐printed phantoms in a spectrum of skin tones can be used to determine the accuracy of SI tracking under treatment‐like conditions. All SI systems showed decreased accuracy with darker skin‐toned phantoms, which increased with an obstructed camera view.

## 1 INTRODUCTION

Surface imaging (SI) can be used to monitor intrafraction motion during stereotactic radiosurgery (SRS).^1^, ^2^, ^3^, ^4^ Surface guided radiotherapy (SGRT) for SRS utilizes optical imaging to monitor motion by tracking the patient's face, which is visible through an open face mask. SI can detect submillimeter patient motion and allow for gating of the radiation treatment to ensure treatment only when the patient is in the desired position.^1^, ^2^

While SI can be highly accurate at reporting intrafraction motion, it demonstrates decreased accuracy in reporting under certain conditions, such as when a camera pod is blocked by the gantry. An SI system was also previously reported to have a statistically significant difference in the median magnitude offset for patients with darker skin tones.^5^ Due to patient skin tone, AAPM Task Group 302 recommends, when possible, testing the impact of surface color on localization accuracy utilizing phantoms with light and dark colors.^6^ At the time of this study, there were no commercially available SI phantoms with different skin tones for SI system testing.

The purpose of this study was to develop a method to investigate the impact of skin tone on the performance of SI systems with the goal of quantifying the difference in localization accuracy across a spectrum of skin tones. While the impact of skin tone has been previously reported for patients receiving SRS, the intent of this study was to quantify the baseline localization accuracy of SI systems with respect to skin tone in SRS treatment‐like conditions utilizing stationary phantoms with multiple skin tones.

## 2 METHODS AND MATERIALS

### 2.1 Phantoms

**FIGURE 1:**
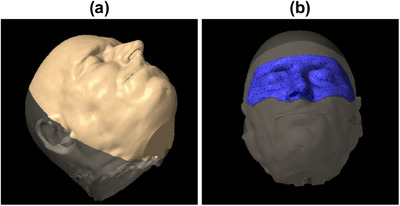
Rendering of the structures in the treatment planning system showing (a) the anterior surface used to generate the 3D printing instructions and (b) the region‐of‐interest for the surface imaging systems.

To design an anthropomorphic phantom that could be made publicly available, images from the Visible Human Project provided courtesy of the United States National Library of Medicine were used. The head CT was downloaded from https://data.lhncbc.nlm.nih.gov/public/Visible‐Human/Additional‐Head‐Images/MR_CT_DICOM/CAT/index.html. The DICOM tags Study Description, Image Comments, Patient Birth Date, and Patient ID were modified to facilitate import into the Eclipse treatment planning system (Varian Medical Systems, Palo Alto, CA, USA). After the external body contour was created, it was copied and cropped at the coronal plane passing through the ears to extract the anterior surface. The external body contour and the anterior surface are shown in Figure 1. Using the Eclipse Scripting Application Programming Interface, the three‐dimensional triangle mesh representing the anterior surface was exported in STL file format.

**FIGURE 2:**
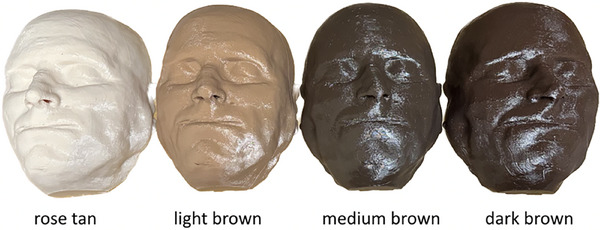
Phantoms 3D printed with PETG filament in four skin tones using a CT scan from the National Institutes of Health (NIH) Visible Human Project. PETG, polyethylene terephthalate glycol.

The STL file was imported into 3D Slicer software^7^ (IdeaMaker, Raise3D, Stafford, TX) to create a gcode file. The model was processed using the “ultra‐high quality” print setting, which included a layer height of 0.1 mm, 25% infill density, 120 mm/s infill speed, and two outer shells. No raft or support structures were needed. Phantoms were 3D printed using polyethylene terephthalate glycol (PETG) filament in each of four colors designed specifically to match skin tones: rose tan, light brown, medium brown, and dark brown (3DUniverse, Chicago, IL). The print time for each phantom was 29 h and 10 min per phantom, and no post‐processing was needed to smooth the surface. The four phantoms are shown in Figure 2.

### 2.2 Surface imaging systems

Three SI systems from two vendors were evaluated. Two systems were AlignRT (VisionRT, London, UK) software version 6.3.1 having different cameras. One of the systems had generation 4 cameras, and the other had generation 5 cameras. The AlignRT systems were located at the University of Michigan. The third system was IDENTIFY (Varian Medical Systems, Palo Alto, CA, USA), located at the University of Alabama at Birmingham. Two different software versions were evaluated for IDENTIFY, v.2.3 and v.3.0. The four system/software combinations are summarized in Table 1. All systems were installed in the standard configuration in vaults with a TrueBeam linear accelerator (Varian Medical Systems, Palo Alto, CA, USA) and were calibrated according to vendor procedures. The primary aim of this work was to develop methods to assess the impact of skin tone on the accuracy of intrafraction motion monitoring for frameless intracranial SRS, not to intercompare the SGRT systems or comment on the suitability of specific systems for SRS application. Therefore, prior to any data collection, the authors agreed to de‐identify the systems in this report. The four system/software combinations were labeled A, B, C, and D in random order. The authors will share the system identification upon request.

**TABLE 1:** The vendor, model, software version, and location of the SI‐systems utilized in this study.

Model	Vendor	Software version	Location
AlignRT—Generation 4 cameras	VisionRT, London, UK	6.3.1	University of Michigan
AlignRT—Generation 5 cameras	VisionRT, London, UK	6.3.1	University of Michigan
IDENTIFY	Varian Medical Systems, Palo Alto, CA, USA	2.3	University of Alabama at Birmingham
IDENTIFY	Varian Medical Systems, Palo Alto, CA, USA	3.0	University of Alabama at Birmingham

Abbreviation: SI, surface imaging.

Both systems use a similar camera geometry consisting of three camera pods mounted above the isocenter. In the IEC room coordinate system, two of the pods are located symmetrically on the *x*‐axis, and the third is located on the negative side of the *z*‐axis. The geometry is such that the gantry blocks one of the pods when it is in the range of 260–350 degrees and 10–100 degrees.

Both systems use a region‐of‐interest (ROI) to define the area of the external body contour to monitor for offsets. For patients in a thermoplastic mask, there is typically an open area around the nose and eyes. The size of the open area is vendor and mask model dependent. We created an ROI based on a mask system intended for SRS, shown in Figure 1. Since the ROI was created from a contour and imported into each SI system, the same ROI was used for all measurements.

The AlignRT system has a skin tone setting with values fair, medium, and dark. The fair, medium, dark, and dark setting were used for the rose tan, light brown, medium brown, and dark brown phantoms, respectively. The IDENTIFY system does not have a skin tone setting. While other commercial systems may have user adjustable parameters such as integration time and gain, neither system in this study has additional user configurable parameters.

### 2.3 Data collection

A treatment plan was created to move the gantry and table positions and to create beam‐on and beam‐off times. The plan was comprised of beams at table angles 0, 45, 90, 315, and 270 degrees and gantry angles 0, 50, and 310 degrees. At gantry angles 50 and 310 degrees, one of the camera pods is obstructed by the gantry. For non‐zero table angles, the non‐zero gantry angle that would result in patient collision was excluded. The gantry‐table angle combinations and associated camera views are shown in Table 2. Each beam had secondary collimator jaws set to 3 cm × 3 cm and a closed multi‐leaf collimator. The monitor units were selected such that the beam was on for 15 s. For each phantom, the plan was loaded, the SI system was used to align the phantom to within 1 mm/1 degree of the planned position, an SI‐reference surface was acquired, and the plan was delivered. To confirm that the phantom did not move during table motion, after all positions given in Table 2 were complete, the initial position (table and gantry at zero) was repeated. For each system of interest, the plan was delivered three times on separate days. The ambient room illumination for all systems was set using a dimmer switch adjusted to a standard marked position in the middle of the range, which was the illumination condition used when the systems were calibrated.

**TABLE 2:** Gantry‐table angle combinations and corresponding camera view.

Table angle	Gantry angle	Camera view
0	0	3‐camera
	50	2‐camera (+x camera location obstructed)
	310	2‐camera (‐x camera location obstructed)
45	0	3‐camera
	310	2‐camera (‐x camera location obstructed)
90	0	3‐camera
	310	2‐camera (‐x camera location obstructed)
315	0	3‐camera
	50	2‐camera (+x camera location obstructed)
270	0	3‐camera
	50	2‐camera (+x camera location obstructed)

### 2.4 Data analysis

Both systems have the capability to export the reported offsets (e.g., the SI reported translational and rotational position relative to the reference position) to text files. At each table/gantry angle combination, the translational offsets along each direction recorded over the middle 5 s (from 5 s after beam‐on to 10 s after beam‐on) of each beam delivery (gantry‐table angle combination) were extracted from the text file. The beam‐on and beam‐off times were obtained from DICOM treatment records created by the treatment delivery system. Synchronizing the SI system offsets with beam delivery ensured that for the extracted offsets, the gantry and table were not in motion. The average offset vector and offset magnitude were calculated given by

(1) $$\bar{\mathrm{Lat}}=\frac{1}{\sum N_{j}}\;\sum_{j=1}^{3}\sum_{i=1}^{N_{j}}\mathrm{La}t_{ij}$$

(2) $$\bar{\mathrm{Vrt}}=\frac{1}{\sum N_{j}}\;\sum_{j=1}^{3}\sum_{i=1}^{N_{j}}\mathrm{Vr}t_{ij}$$

(3) $$\bar{\mathrm{Lng}}=\frac{1}{\sum N_{j}}\;\sum_{j=1}^{3}\sum_{i=1}^{N_{j}}\mathrm{Ln}g_{ij}$$

(4) $$\mathrm{Ma}g_{ij}=\sqrt{\mathrm{La}{t_{ij}}^{2}+\mathrm{Vr}{t_{ij}}^{2}+\mathrm{Ln}{g_{ij}}^{2}}\;$$

(5) $$\bar{\mathrm{Mag}}=\frac{1}{\sum N_{j}}\;\sum_{j=1}^{3}\sum_{i=1}^{N_{j}}\mathrm{Ma}g_{ij}$$

where Lat*_ij_*, Vrt*_ij_*, and Lng*_ij_* are the lateral, vertical, and longitudinal offsets reported by the SI system at time *i* and delivery session *j*, and *N_j_* is the number of offsets reported during the 5 s period at the table and gantry angle of interest for session *j*. The magnitude of the offset difference relative to the rose tan phantom at each table and gantry angle is given by

(6) $$\bar{\Delta {\mathrm{Mag}}_{\mathrm{RoseTan}}^{\mathrm{Phantom}}}=\frac{1}{3}\;\sum_{j=1}^{3}\left({\bar{\mathrm{Mag}}}_{j,\mathrm{Phantom}}-{\bar{\mathrm{Mag}}}_{j,\mathrm{RoseTan}}\right)$$

was also calculated.

## 3 RESULTS

**FIGURE 3:**
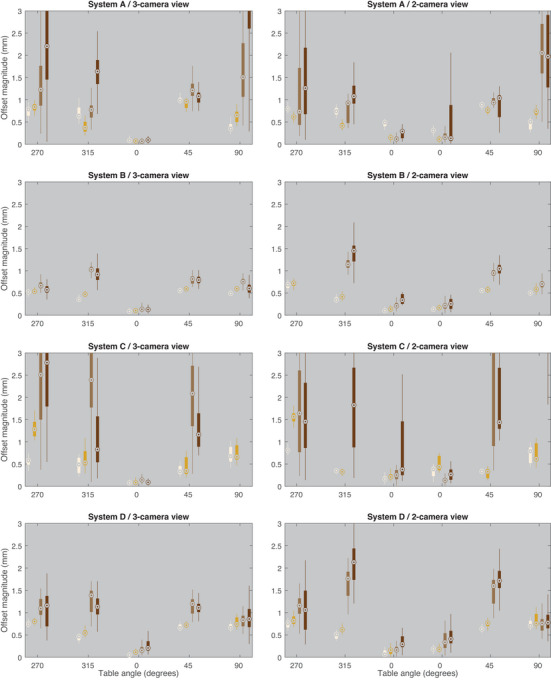
Box‐whisker plots of the phantom offset from the reference position for three sessions at each table angle of the rose tan, light brown, medium brown, and dark brown phantoms. For the 2‐camera views at table angle 0, there are two box‐whisker shown because a camera can be blocked at gantry angle 50 or gantry angle 310. For table angles missing a box‐whisker, the system either reported offsets > 3 mm or did not report any offset. See Table 2 for quantitative values.

**FIGURE 4:**
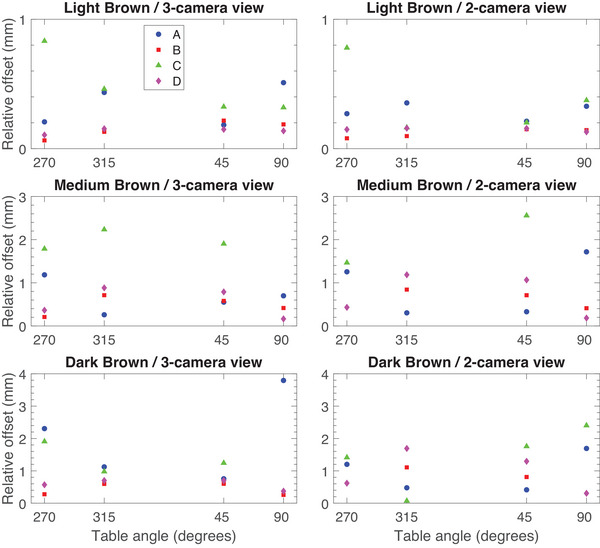
The mean offset relative to the rose tan phantom at non‐zero table angles for systems A (blue circles), B (red squares), C (green triangles), and D (purple diamonds) for 3‐ and 2‐camera views. For table angles missing a point, the system did not report offsets at that table angle.

The median and inter‐quartile ranges of the offset magnitudes (Equation 4) are shown in Figure 3 and summarized in Table 3. Note that at a table angle of 0 degrees, two gantry configurations lead to a 2‐camera view (gantry 50 and 310); therefore, both values are presented. Entries with “no data” indicate that the SI system was unable to resolve the location of the phantom, and no values were recorded. Note that for the SI system with a skin tone setting, the medium dark and dark phantoms were only measured with the dark skin tone setting due to the inability to capture a reference surface with the fair or medium skin tone settings. For the rose tan and light brown phantoms, the settings of fair and medium were chosen, respectively, as the most representative setting for the phantom. The difference in offset magnitude relative to the rose tan phantom (Equation 5) at each table angle is shown in Figure 4. System A showed consistent performance across different phantoms, with median offsets generally below 1.0 for unobstructed views, except for the dark brown phantom, which had higher offsets at certain angles. System B demonstrated similar trends, with slightly lower offsets for the rose tan and light brown phantoms, but failed to report data for the med brown and dark brown phantoms at some angles. System C exhibited higher variability, particularly for the med brown and dark brown phantoms, with some angles showing no data. System D maintained relatively stable offsets across phantoms, with occasional higher values for the med brown and dark brown phantoms. Overall, the unobstructed views tended to yield lower offsets compared to obstructed views.

**TABLE 3:** The median and interquartile range of the SI‐reported offsets of each phantom with either an unobstructed (3‐camera) or obstructed (2‐camera) view.

System ID			Table angle					
	Phantom	Cameras	270	315	0		45	90
A	RoseTan	3	0.8 (0.2)	0.6 (0.2)	0.1 (0.1)		1.0 (0.1)	0.4 (0.1)
		2	0.8 (0.1)	0.7 (0.2)	0.5 (0.1)	0.3 (0.1)	0.9 (0.1)	0.5 (0.2)
	LtBrown	3	0.8 (0.1)	0.4 (0.2)	0.1 (0.0)		1.0 (0.2)	0.6 (0.2)
		2	0.6 (0.1)	0.4 (0.1)	0.1 (0.1)	0.1 (0.1)	0.8 (0.1)	0.7 (0.1)
	MedBrown	3	1.2 (0.9)	0.8 (0.3)	0.1 (0.0)		1.2 (0.3)	1.5 (1.2)
		2	0.7 (1.3)	0.9 (0.5)	0.1 (0.1)	0.2 (0.1)	0.9 (0.1)	2.1 (1.1)
	DkBrown	3	2.2 (1.9)	1.6 (0.5)	0.1 (0.1)		1.1 (0.2)	4.5 (3.2)
		2	1.3 (1.5)	1.1 (0.4)	0.3 (0.2)	0.1 (0.8)	1.0 (0.5)	2.0 (1.6)
B	RoseTan	3	0.5 (0.1)	0.4 (0.1)	0.1 (0.0)		0.6 (0.0)	0.5 (0.1)
		2	0.7 (0.1)	0.3 (0.1)	0.1 (0.1)	0.1 (0.0)	0.6 (0.0)	0.5 (0.0)
	LtBrown	3	0.5 (0.1)	0.5 (0.1)	0.1 (0.0)		0.6 (0.0)	0.6 (0.0)
		2	0.7 (0.1)	0.4 (0.1)	0.1 (0.0)	0.2 (0.0)	0.6 (0.0)	0.6 (0.1)
	MedBrown	3	0.7 (0.1)	1.0 (0.1)	0.1 (0.1)		0.8 (0.2)	0.8 (0.1)
		2	*No data*	1.1 (0.2)	0.2 (0.1)	0.2 (0.1)	0.9 (0.1)	0.7 (0.1)
	DkBrown	3	0.6 (0.1)	0.9 (0.3)	0.1 (0.1)		0.8 (0.1)	0.6 (0.2)
		2	*No data*	1.5 (0.4)	0.3 (0.2)	0.3 (0.2)	1.0 (0.2)	*No data*
C	RoseTan	3	0.6 (0.1)	0.5 (0.3)	0.1 (0.0)		0.3 (0.2)	0.7 (0.3)
		2	0.8 (0.1)	0.4 (0.0)	0.2 (0.1)	0.4 (0.2)	0.3 (0.1)	0.8 (0.4)
	LtBrown	3	1.3 (0.3)	0.5 (0.3)	0.1 (0.1)		0.3 (0.4)	0.7 (0.3)
		2	1.6 (0.2)	0.3 (0.1)	0.2 (0.1)	0.4 (0.3)	0.3 (0.2)	0.6 (0.4)
	MedBrown	3	2.5 (2.2)	2.4 (1.3)	0.1 (0.1)		2.1 (1.4)	*No data*
		2	1.6 (1.8)	*No data*	0.3 (0.2)	0.1 (0.1)	3.3 (3.4)	5.0 (1.0)
	DkBrown	3	2.8 (2.0)	0.8 (1.0)	0.1 (0.1)		1.2 (0.8)	*No data*
		2	1.5 (1.5)	1.8 (1.8)	0.4 (1.2)	0.3 (0.2)	1.4 (1.4)	3.6 (0.8)
D	RoseTan	3	0.7 (0.1)	0.5 (0.1)	0.0 (0.0)		0.7 (0.1)	0.7 (0.1)
		2	0.8 (0.2)	0.5 (0.1)	0.1 (0.1)	0.2 (0.1)	0.6 (0.1)	0.7 (0.1)
	LtBrown	3	0.8 (0.0)	0.5 (0.1)	0.1 (0.0)		0.7 (0.1)	0.7 (0.2)
		2	0.8 (0.2)	0.6 (0.1)	0.2 (0.1)	0.2 (0.1)	0.8 (0.1)	0.8 (0.3)
	MedBrown	3	1.1 (0.4)	1.4 (0.5)	0.2 (0.1)		1.2 (0.3)	0.8 (0.2)
		2	1.2 (0.3)	1.8 (0.5)	0.2 (0.1)	0.3 (0.3)	1.6 (0.5)	0.8 (0.3)
	DkBrown	3	1.2 (0.7)	1.1 (0.4)	0.2 (0.2)		1.1 (0.2)	0.9 (0.4)
		2	1.1 (0.9)	2.1 (0.7)	0.3 (0.2)	0.4 (0.3)	1.7 (0.4)	0.8 (0.3)

*Note*: “No data” indicates that the SI system failed to report values at the designated table angle

Abbreviation: SI, surface imaging.

Across all measurements for all systems and phantoms, the mean (standard deviation) of the offsets at gantry and table zero after all measurements given in Table 2 were complete was −0.1 (0.05), 0.04 (0.1), and −0.1 (0.05) mm in the lateral, longitudinal, and vertical directions, respectively. These results confirm that the phantoms did not move during the measurements.

The raw data offsets extracted from the text files are given in the Supporting Information.

## 4 DISCUSSION

In the present study, the SI systems tested showed an increase in the reported offset of the stationary phantoms when observed at a non‐zero couch angle, consistent with previous studies on intrafraction patient motion.^5^ The magnitude of these offsets increased further when a camera pod was obstructed and could increase by up to 5 mm for one system or render the SI system unable to resolve the location of the medium brown and dark brown phantoms, as shown in Table 3. The false reports of motion compromise the utility of SI for SRS, where action levels to stop treatment are typically 1 mm. As shown in Figure 4, relative to the rose tan phantom, the light, medium, and dark brown phantoms show false offsets of increasing magnitude with regard to skin tone for all systems.

One factor that may influence SI system performance is room illumination. The measurements reported here used room illumination typical of clinical use, which is that used for system calibration. However, it is possible that brighter or dimmer illumination could improve performance. The approach reported here is ideal for exploring the effect of room illumination, as well as any other factors that might impact SI system performance.

In a previous study, patient data for 370 patients receiving SRS were monitored with the IDENTIFY system, and reported offsets at non‐zero couch angles, with and without an obstructed camera view, were compared between Black and White patients and found to be statistically significant.^5^ Note that AlignRT was previously studied for equitable treatment between Black and White patients, and although results were found to not be statistically significant, the data were not analyzed for obstructed camera views where the largest variances between skin tones are observed.^8^ While monitoring and analyzing intrafraction motion for large cohorts of patients highlighted performance issues for different patient populations, characterizing system performance is complicated by the inclusion of patient motion and variations in patient characteristics, such as the patient‐specific ROI. To remove these factors and assess the baseline performance of the systems, anthropomorphic phantoms created from the same CT dataset with identical regions of interest were utilized.

At the time of this study, no commercial phantoms were available for purchase following the TG‐302 recommendation of testing SI systems utilizing light and dark skin phantoms. Many commercial phantoms used for SRS commissioning have either a non‐skin tone color and/or glossy exteriors, which make them unsatisfactory for SI testing. While utilizing a custom phantom that could be easily accessible by clinics utilizing SI would be ideal, the lack of commercial options required the creation of custom phantoms for testing. The use of 3D printing was selected due to the relatively low cost and availability of commercial skin tone filaments to create multiple phantoms for testing. In a study by Kapoor et al., skin tone cloths draped on a pelvis phantom were used to evaluate SI camera sensitivity to skin tone.^9^ While this allowed for a broad spectrum of skin tones for evaluation, 3D printing was utilized in the current study to utilize an anthropomorphic phantom with a representative SRS ROI. To ensure that this methodology can be widely used, the 3D printing file, DICOM files containing the CT images and structure set used to create the 3D printer file, and the plan used for data collection are available at https://github.com/UAB‐RO/Head‐to‐head_phantom.

This study highlights the importance of evaluating technology for equitable performance across all patient populations. When purchasing and commissioning new technologies, the potential to cause or contribute to health disparities should be evaluated and accounted for, as highlighted by Moncion et al., who created a framework for quantifying the risk of technology‐driven disparities.^10^ Vendors should be encouraged to disclose performance data for diverse patient cohorts to assist clinics with this assessment. Outcomes of this assessment may include creating a supplemental workflow to address the suboptimal performance (e.g., radiographic imaging) and ensure equitable treatment of all patients.

## 5 CONCLUSIONS

Due to a lack of commercial phantoms available with a spectrum of skin tones, 3D printing is a viable alternative to assess the accuracy of SI systems using publicly available CT datasets and the provided printing files. The SI systems tested showed decreased accuracy when tracking anthropomorphic phantoms with darker skin tones when compared to light skin toned phantoms, which is consistent with data collected comparing SI‐reported offsets for patients monitored during SRS. This work highlights the importance of following the TG‐302 recommendations to assess SI systems for accuracy across a spectrum of skin tones to fully characterize SI systems before clinical implementation, during routine quality assurance, and following system updates. Alternative or supplemental techniques to assess intrafraction motion (e.g., radiographic imaging) may be needed due to sub‐optimal performance of SI imaging for patients with darker skin.

## AUTHOR CONTRIBUTION

Elizabeth L. Covington made substantial contributions to the conception and design of the work; the acquisition, analysis, and interpretation of data for the work; drafting the work and revising it critically for important intellectual content; gave final approval of the version to be published; and agrees to be accountable for all aspects of the work in ensuring that questions related to the accuracy or integrity of any part of the work are appropriately investigated and resolved. Dennis N. Stanley made substantial contributions to the acquisition, analysis, and interpretation of data for the work; drafting the work and revising it critically for important intellectual content; gave final approval of the version to be published; and agrees to be accountable for all aspects of the work in ensuring that questions related to the accuracy or integrity of any part of the work are appropriately investigated and resolved. Ashanti Lawson made substantial contributions to the acquisition, analysis, and interpretation of data for the work; drafting the work and revising it critically for important intellectual content; gave final approval of the version to be published; and agrees to be accountable for all aspects of the work in ensuring that questions related to the accuracy or integrity of any part of the work are appropriately investigated and resolved. Scott Hadley made substantial contributions to the acquisition, analysis, and interpretation of data for the work; drafting the work and revising it critically for important intellectual content; gave final approval of the version to be published; and agrees to be accountable for all aspects of the work in ensuring that questions related to the accuracy or integrity of any part of the work are appropriately investigated and resolved. Alexander Moncion made substantial contributions to the acquisition, analysis, and interpretation of data for the work; drafting the work and revising it critically for important intellectual content; gave final approval of the version to be published; and agrees to be accountable for all aspects of the work in ensuring that questions related to the accuracy or integrity of any part of the work are appropriately investigated and resolved. Rodney J. Sullivan made substantial contributions to the acquisition, analysis, and interpretation of data for the work; drafting the work and revising it critically for important intellectual content; gave final approval of the version to be published; and agrees to be accountable for all aspects of the work in ensuring that questions related to the accuracy or integrity of any part of the work are appropriately investigated and resolved. John B. Fiveash made substantial contributions to the acquisition, analysis, and interpretation of data for the work; drafting the work and revising it critically for important intellectual content; gave final approval of the version to be published; and agrees to be accountable for all aspects of the work in ensuring that questions related to the accuracy or integrity of any part of the work are appropriately investigated and resolved. Richard A. Popple made substantial contributions to the conception and design of the work; the acquisition, analysis, and interpretation of data for the work; drafting the work and revising it critically for important intellectual content; gave final approval of the version to be published; and agrees to be accountable for all aspects of the work in ensuring that questions related to the accuracy or integrity of any part of the work are appropriately investigated and resolved.

## CONFLICT OF INTEREST STATEMENT

The IDENTIFY system was provided to The University of Alabama at Birmingham as part of a product evaluation agreement with Varian Medical Systems.

## Supporting information



Supporting Information
